# Assessing ChatGPT’s Capability for Multiple Choice Questions Using RaschOnline: Observational Study

**DOI:** 10.2196/46800

**Published:** 2024-08-08

**Authors:** Julie Chi Chow, Teng Yun Cheng, Tsair-Wei Chien, Willy Chou

**Affiliations:** 1 Department of Pediatrics Chi Mei Medical Center Tainan Taiwan; 2 Department of Pediatrics School of Medicine, College of Medicine Chung Shan Medical University Taichung Taiwan; 3 Department of Emergency Medicine Chi Mei Medical Center Tainan Taiwan; 4 Department of Statistics Coding Data Analytics Tainan Taiwan; 5 Department of Physical Medicine and Rehabilitation Chi Mei Medical Center Tainan Taiwan; 6 Department of Leisure and Sports Management Far East University Tainan Taiwan

**Keywords:** RaschOnline, ChatGPT, multiple choice questions, differential item functioning, Wright map, KIDMAP, website tool, evaluation tool, tool, application, artificial intelligence, scoring, testing, college, students

## Abstract

**Background:**

ChatGPT (OpenAI), a state-of-the-art large language model, has exhibited remarkable performance in various specialized applications. Despite the growing popularity and efficacy of artificial intelligence, there is a scarcity of studies that assess ChatGPT’s competence in addressing multiple-choice questions (MCQs) using KIDMAP of Rasch analysis—a website tool used to evaluate ChatGPT’s performance in MCQ answering.

**Objective:**

This study aims to (1) showcase the utility of the website (Rasch analysis, specifically RaschOnline), and (2) determine the grade achieved by ChatGPT when compared to a normal sample.

**Methods:**

The capability of ChatGPT was evaluated using 10 items from the English tests conducted for Taiwan college entrance examinations in 2023. Under a Rasch model, 300 simulated students with normal distributions were simulated to compete with ChatGPT’s responses. RaschOnline was used to generate 5 visual presentations, including item difficulties, differential item functioning, item characteristic curve, Wright map, and KIDMAP, to address the research objectives.

**Results:**

The findings revealed the following: (1) the difficulty of the 10 items increased in a monotonous pattern from easier to harder, represented by logits (–2.43, –1.78, –1.48, –0.64, –0.1, 0.33, 0.59, 1.34, 1.7, and 2.47); (2) evidence of differential item functioning was observed between gender groups for item 5 (*P*=.04); (3) item 5 displayed a good fit to the Rasch model (*P*=.61); (4) all items demonstrated a satisfactory fit to the Rasch model, indicated by Infit mean square errors below the threshold of 1.5; (5) no significant difference was found in the measures obtained between gender groups (*P*=.83); (6) a significant difference was observed among ability grades (*P*<.001); and (7) ChatGPT’s capability was graded as A, surpassing grades B to E.

**Conclusions:**

By using RaschOnline, this study provides evidence that ChatGPT possesses the ability to achieve a grade A when compared to a normal sample. It exhibits excellent proficiency in answering MCQs from the English tests conducted in 2023 for the Taiwan college entrance examinations.

## Introduction

### Background

ChatGPT is an advanced language model, which stands for Chat Generative Pretrained Transformer [1]. Its primary function is to generate text that mimics human language based on a given prompt or context [1,2]. This state-of-the-art model has been trained using an extensive amount of text data available on the internet, enabling it to understand and produce text on a diverse range of subjects and in various language styles [3-5].

ChatGPT is a highly versatile language model that has found numerous applications [1]. One such significant use is text generation, which could revolutionize content creation, including academic publications [4,5]. With the ever-growing sophistication of language models, such as ChatGPT, differentiating between text produced by humans and that generated by artificial intelligence (AI) will become increasingly difficult [3]. ChatGPT can respond to user prompts to perform a variety of tasks, such as answering questions, composing essays, writing poems and love letters, generating computer code, and even creating business plans. Furthermore, it can also solve complex problems, including those in math or physics, among other fields [6-8].

### Assessing ChatGPT’s Capacity for Multiple-Choice Questions

Korn and Kelly [9] have raised serious doubts about the reliability and fairness of ChatGPT, echoing concerns voiced in the popular press regarding the chatbot’s tendency to disseminate misinformation. The authors caution that ChatGPT may not always provide accurate information [9], and there are fears that it could be manipulated to spread false information [10] or produce “deepfakes” [11].

Research on medical question answering has previously evaluated ChatGPT’s performance on specific tasks [12]. For example, Jin et al [13] achieved 68.1% accuracy in answering yes-or-no questions from PubMed abstracts, while ChatGPT performed with accuracy rates of 64.4% and 57.8% on 2 data sets from the United States Medical Licensing Examination (USMLE) [12]. ChatGPT also achieved high scores on breast cancer screening prompts [14] and Kawasaki disease prompts [3,13,15-21]. A total of 2 pediatricians’ assessments indicated that ChatGPT’s overall performance corresponded to a grade of C in a range from A to E, with average scores of –0.89 logits and 0.90 logits (=log odds), respectively [22].

Recent research findings indicate that ChatGPT has shown remarkable precision in answering questions related to the US Certified Public Accountant exam and the US bar examination [23,24]. Additionally, in the field of medicine, ChatGPT has met the required standards for the USMLE [14,25]. While there are still obstacles to overcome when applying ChatGPT to clinical medicine [26-28], it has demonstrated satisfactory performance in English examinations [29]. However, Ha and Yaneva [30] reported low accuracy rates for medical multiple-choice questions (MCQs). In this study, we were motivated to determine ChatGPT’s grade (eg, A, B, C, or D) in answering MCQs against the study [22] with low accuracy for MCQs.

### Rasch Model Applied to This Study

In ChatGPT, there are 2 types of prompts: MCQs [30] and open-ended (OE) [3,14]. The OE format of ChatGPT is more subjective than the MCQs. MCQs can be objectively evaluated by observing the correct and incorrect answers to each item. The Rasch model [31] is suitable for analyzing dichotomous responses (ie, correct and incorrect answers). Otherwise, the Rasch rating scale model (RSM) [32] can be applied. Nonetheless, a study using Rasch analysis to examine the capability of ChatGPT has not yet been published in the literature. Therefore, it is necessary to demonstrate the use of Rasch analysis in assessing ChatGPT’s capability based on MCQs with correct and incorrect answers (ie, dichotomous responses in Rasch analysis).

### Features of Rasch Analysis

#### Overview

Rasch analysis is a statistical method that evaluates the performance of individuals on tests or assessments. By applying this technique to ChatGPT [1], researchers can assess the quality of its responses and pinpoint areas that may require improvement [33]. Below are some of the features of Rasch analysis that can be applied to evaluate the performance of ChatGPT.

#### Item Difficulty

Rasch analysis can provide valuable insights into the difficulty level of each prompt or question presented to ChatGPT. This information can be used to pinpoint areas where ChatGPT may face challenges (such as when presented with difficult questions) or perform well (such as when presented with easier questions) [34].

#### Person Ability

By analyzing ChatGPT’s responses to prompts or questions, Rasch analysis can measure its ability level. This evaluation can offer valuable information about ChatGPT’s overall performance and highlight areas where enhancements may be necessary [34].

#### Item Fit Statistics

Item fit statistics are generated through Rasch analysis to evaluate the degree to which each prompt or question aligns with the overall model. This analysis can be used to identify items that require revision or removal from the assessment [35-37].

#### Differential Item Functioning

Differential item functioning (DIF) can be identified by Rasch analysis when different groups of individuals (such as males and females or individuals from diverse cultural backgrounds) respond differently to the same item [38], for example, a specific item may be preferred by men or women based on DIF analysis. By detecting DIF, Rasch analysis can flag potentially biased items and facilitate the improvement of assessment fairness [39].

A total of 5 visualizations are frequently applied to present item features and person measures, including the distribution of item difficulties (DID) [33], DIF [38], item characteristic curve (ICC) [40,41], Wright map (namely, item-person map) [33], and KIDMAP [42]. A forest plot [43] can be used to integrate DID and DIF for a better understanding of item characteristics.

### Study Aims

The study objectives were to (1) demonstrate the use of website Rasch analysis (namely, RaschOnline [44]) and (2) determine the ChatGPT’s grade compared to a normal sample.

## Methods

### Data Source

In this study, 300 simulated participants responded to 10 items from Taiwan college entrance examinations for the year 2023 (Table 1 and Multimedia Appendix 1) with 2-response categories [45] (eg, 0 and 1 for incorrect and correct answers) and were analyzed according to item difficulty (with a logit unit from –2.5 to 2.5; eg, –2.43, –1.78, –1.48, –0.64, –0.1, 0.33, 0.59, 1.34, 1.7, and 2.47 logits) in the Rasch model based on the normal distribution of person measures (Multimedia Appendix 2); see MP4 video [46] and the approach of simulation generation [47] in RaschOnline [44] about the way to conduct this study.

Each item in Table 1 was prompted. Answers from ChatGPT were gathered and scored on a binary scale with 301 people answering the 10 items (Table 1).

The 301 simulated participants were randomly divided into 2 groups based on gender. There were 5 grades assigned based on the person measures (eg, >3.0, >1.5, >–1.5, >–3.0, and ≤–3.0 logits).

As a final step, the 301 individuals (including the ChatGPT301 student) were analyzed using RaschOnline software [44].

**Table 1:** The 10 items used for examining ChatGPT’s capability^a^.

Answer	Number	Item
A	1	The bus driver often complains about chewing gum found under passenger seats because it is () and very hard to remove. (A) sticky, (B) greasy, (C) clumsy, (D) mighty
C	2	Jesse is a talented model. He can easily adopt an elegant () for a camera shoot. (A) clap, (B) toss, (C) pose, (D) snap
C	3	To draw her family tree, Mary tried to trace her () back to their arrival in North America. (A) siblings, (B) commuters, (C) ancestors, (D) instructor
B	4	Upon the super typhoon warning, Nancy rushed to the supermarket—only to find the shelves almost () and the stock nearly gone. (A) blank, (B) bare, (C) hollow, (D) queer
D	5	Even though Jack said “Sorry!” to me in person, I did not feel any () in his apology. (A) liability, (B) generosity, (C) integrity, (D) sincerity
D	6	My grandfather has astonishing powers of (). He can still vividly describe his first day at school as a child. (A) resolve, (B) faction, (C) privilege, (D) recall
B	7	Recent research has found lots of evidence to () the drug company’s claims about its “miracle” tablets for curing cancer. (A) provoke, (B) counter, (C) expose, (D) convert
A	8	Corrupt officials and misguided policies have () the country’s economy and burdened its people with enormous foreign debts. (A) crippled, (B) accelerated, (C) rendered, (D) ventured
A	9	As a record number of fans showed up for the baseball final, the highways around the stadium were () with traffic all day. (A) choked, (B) disturbed, (C) enclosed, (D) injected
D	10	Studies show that the () unbiased media are in fact often deeply influenced by political ideology. (A) undoubtedly, (B) roughly, (C) understandably, (D) supposedly

^a^The prompt to ChatGPT is described as “which is the correct word to fill in the blank in the sentence following: item content” (see MP4 video [46] about the way to conduct this study).

### Ethical Considerations

In the case of this study comparing the accuracy using ChatGPT in English test for students’ answers to the test, it is important to understand that this kind of research does not involve direct interaction with human participants. The focus here is on the performance of the AI model, and the “subjects” are essentially the algorithms themselves. There is no risk of physical, emotional, or psychological harm to human individuals, and there is no collection of personally identifiable information or any sensitive data from humans. Therefore, the Taiwan Ministry of Health and Welfare provides guidelines for research that is exempt from institutional review board review.

### Rasch Analysis of Item Features and Person Responses Using RaschOnline

RaschOnline [44] based on the Rasch RSM model [32] was used to analyze the data. The multitomous data can therefore be analyzed using RaschOnline [44].

In the Rasch model, the probability and SE of the person estimate can be expressed as equations 1 and 2:



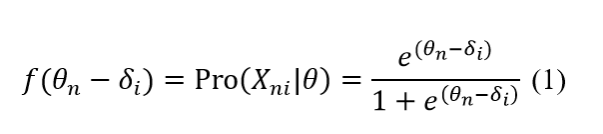





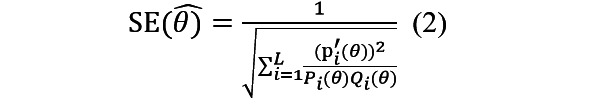



where *θ* and *δ* are defined as person ability and item difficulty, respectively. *L* is the item length. 
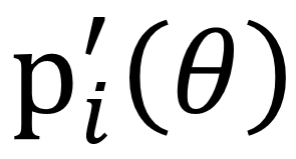
 is the first-order derivative for person *n* with ability *θ* on item *i* in equation 1; *P_i_*(*θ*) is identical to equation 1; *Q_i_*(*θ*) refers to equation 3, as shown below:



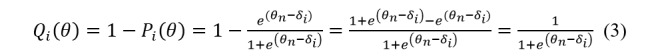



The processes of the first-order derivative for 
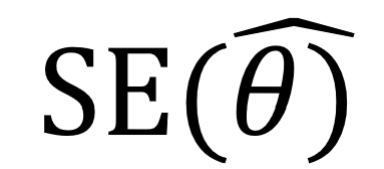
 in equation 2 are described below:



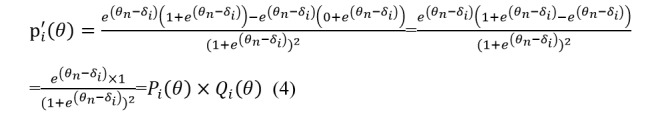



Equation 2 can then be extended to equation 5, indicating that person SE is associated with the inverse of its total variances across all items.



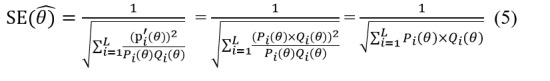



The processes of the first-order derivative for variance (denoted by Var*_ni_*) on (*θ_n_*–*δ_i_*) can also be described based on equation 4 and are shown below



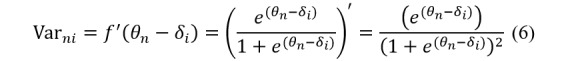



If (*e*^(^*^θn^*^–^*^δi^*^)^) is replaced with 
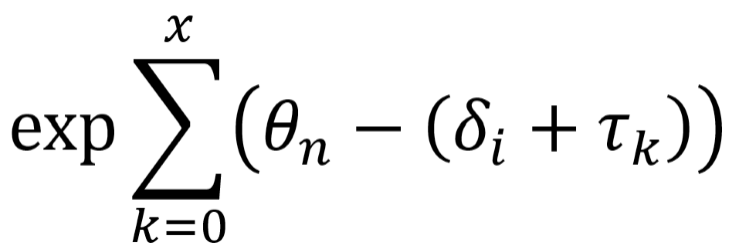
 , the variance for person *n* on item *i* adaptive to the RSM equals the result in equation 6 [48]. Through the Newton-Raphson iteration method [49] and the person estimate and 
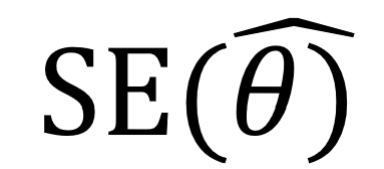
 in equations 1 and 5, RaschOnline [44,50] was programmed and developed.

To visualize item features and individual measures, several visualizations are commonly used, such as DID [33], DIF [38], ICC [40,41], Wright map [51], and KIDMAP [42].

The method of drawing these visualizations refers to the manual of RaschOnline [44] and Multimedia Appendix 3 (how to conduct this study).

### Two Tasks Required to Achieve the Study Goals

#### Demonstrate the Use of RaschOnline (Task 1)

Rasch analysis was used to observe item features and person responses (eg, the determination of grade in ChatGPT performance [22]), and some significant terms in Rasch analysis are defined: (1) DIF [38] analysis was performed to examine whether there are items in favor of a specific group (eg, Female or Male), for example, a specific item might be in favor of female (or male) to be easy in response. Details about DIF are in Multimedia Appendix 3; (2) the ICC [40,41] is a plot of the probability of the examinee answering a question correctly against his or her underlying abilities on the trait being measured [33]. The ICC is based on item response theory: the curve is bounded between 0 and 1, monotonically increases, and is commonly referred to as a logistic function. There is a characteristic curve for each item in a test; (3) Wright map [51] with groups was used to display sample distributions of groups compared to the overall sample of item difficulties and person performance abilities with a log-odds(=logit) unit on a common equal-interval continuum. ANOVA was performed to examine differences in measures between groups (eg, Female and Male); (4) in the KIDMAP [42], individual person performance is assessed using the *z* score (observed×expected÷SD) across items. The *z* scores of items outside the upper limit (>2.0) indicate that the observed responses are significantly higher than those expected or *z* scores (<–2.0) with unexpected responses based on the individual’s ability.

In task 1, the first study goal of the determination of RaschOnline [44] would be achieved.

#### Determine ChatGPT’s Grade Against Normal Sample (Task 2)

Using Rasch analysis, the capability of ChatGPT301 to answer 10-item MCQs from Taiwan college entrance examinations for the year 2023 (Table 1 and Multimedia Appendix 1) can be assessed.

In task 2, the second study goal of determining ChatGPT’s grade compared to a normal sample would be achieved.

### Statistical Tools and Data Analysis

SPSS Statistics (version 22.0; IBM Corp) for Windows and MedCalc (version 9.5.0.0; MedCalc Software) for Windows were used to help perform Rasch analysis. Type I errors were set at a significance level of 0.05.

The 5 visualizations include DID [33], DIF [38], ICC [40,41], Wright map [51], and KIDMAP [42] in tasks 1 and 2 of this study.

**Figure 1:**
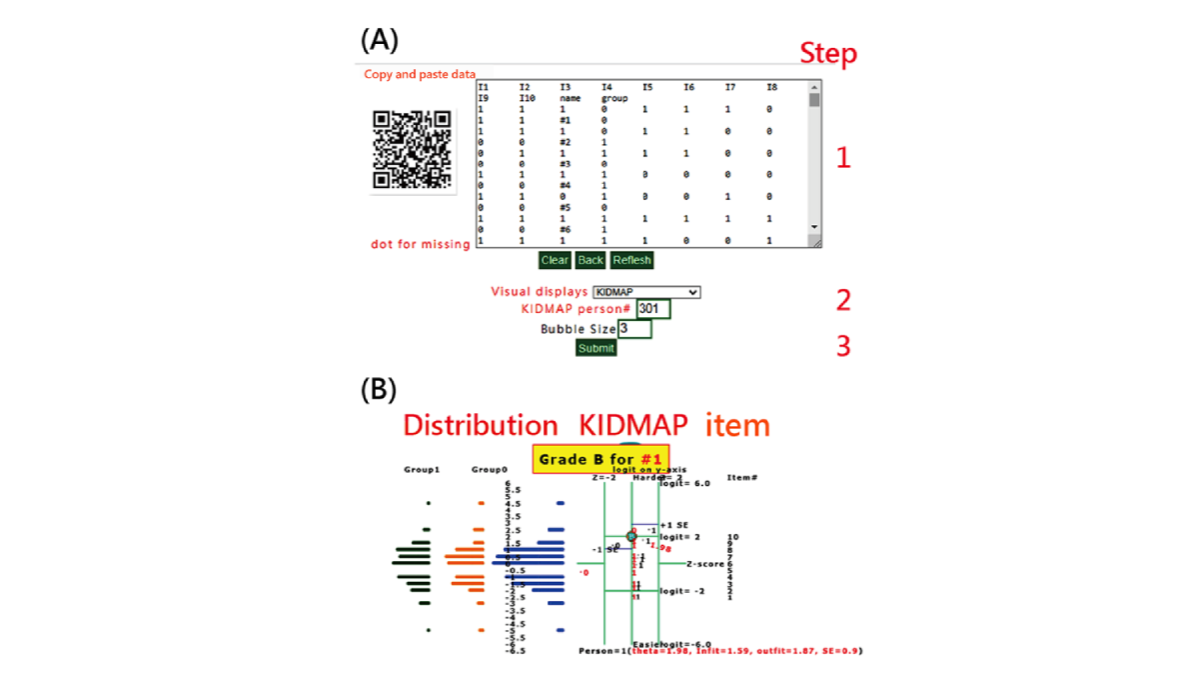
How to execute RaschOnline with the example of KIDMAP (note: (1) data are copied and pasted to the box frame; (2) visual presentation is selected; (3) submission icon is clicked to generate results). (A) Data entry; (B) Data display.

Details about how to conduct this study can be found in the link (MP4) provided in references [46,49] and in Figure 1 (eg, copy and paste data into the box, select visual display, and click on submit icon to draw website visual representations).

## Results

### Demonstrate the Use of RaschOnline (Task 1)

**Figure 2:**
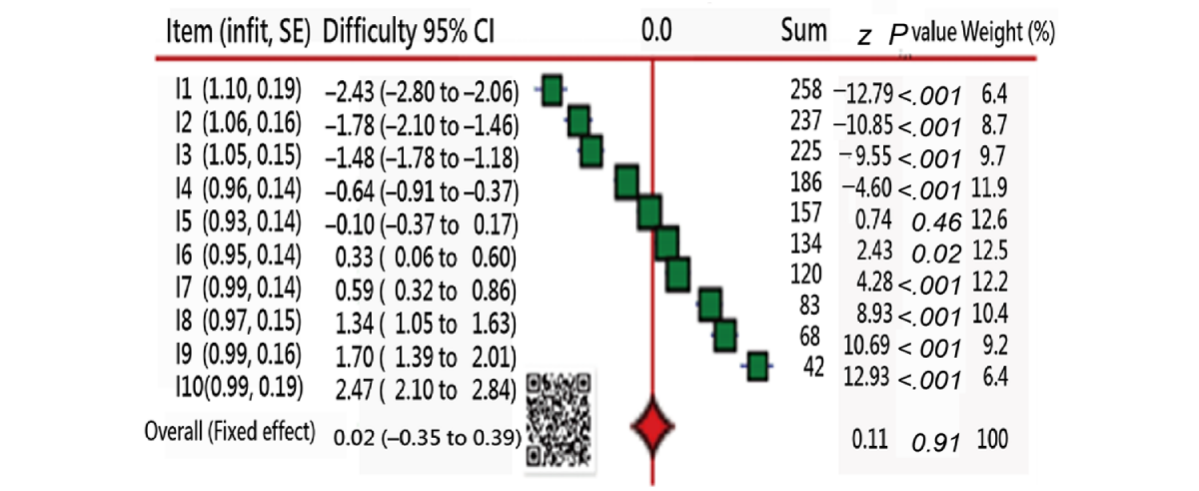
Distribution of item difficulties used in this study.

The DID is shown in Figure 2. All 10 items fit Rasch rather well (ie, Infit meansquares [MNSQs] of all items less than 1.5, as shown in the first column of Figure 2). The reason for fitting the Rasch model is that all data were simulated under the Rasch RSM model. It is stated that item difficulties are from the easiest (left) to the hardest (right) and refer to the summation scores: the easy items will have a higher summation score.

**Figure 3:**
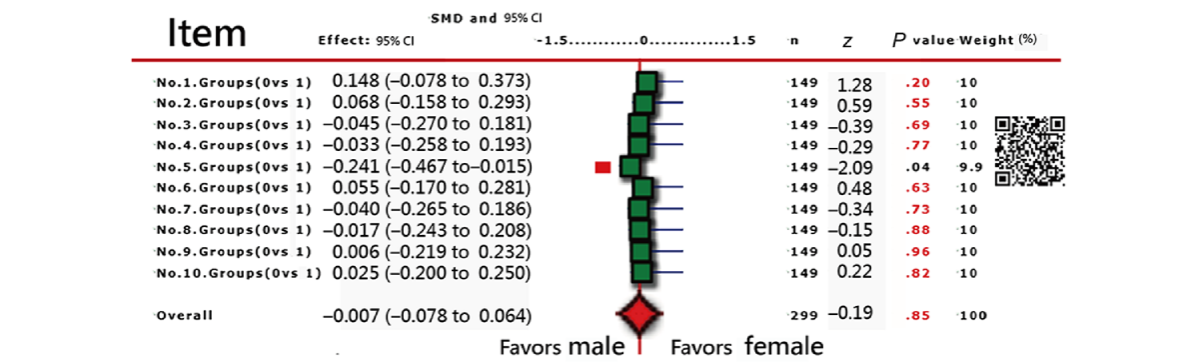
DIF analysis of the 10 items in this study (note: item 5 exhibits a small DIF effect with *P*=.04<.05). DIF: differential item functioning; SMD: standardized mean difference.

The items in Figure 3 are all DIF-free, but item 5 has a slight DIF (*P*=.04). The reason for this is that all responses are generated using a Rasch RSM model, and the gender groups are randomly assigned to each simulated participant.

**Figure 4:**
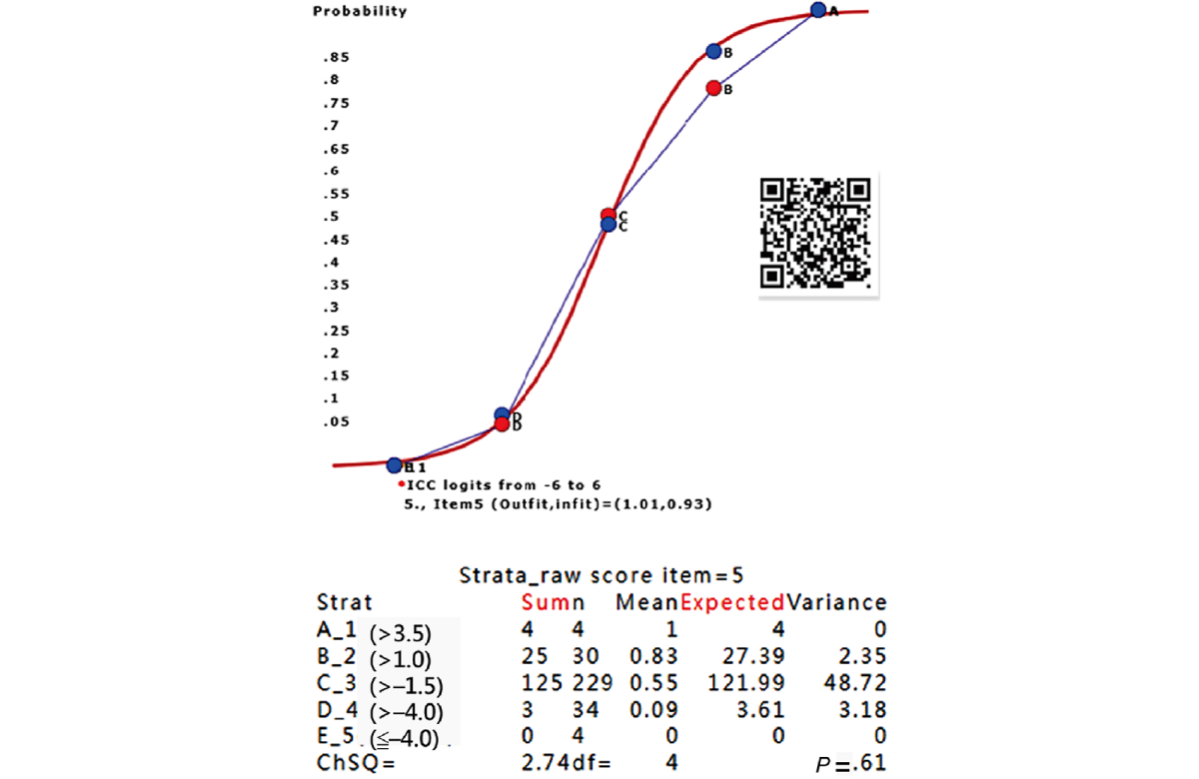
ICC of item 5 fits the Rasch model (*P*=.61). ICC: item characteristic curve.

The ICCs for item 5 are shown in Figure 4. There is a slight deviation from the expected scores in stratum B. Nonetheless, item 5 is still fitted to the Rasch model, with *P*=.61, based on chi-square fit statistics [35].

The first study goal of the demonstration of RaschOnline has been achieved.

### Determine ChatGPT’s Grade Against a Normal Sample (Task 2)

**Figure 5:**
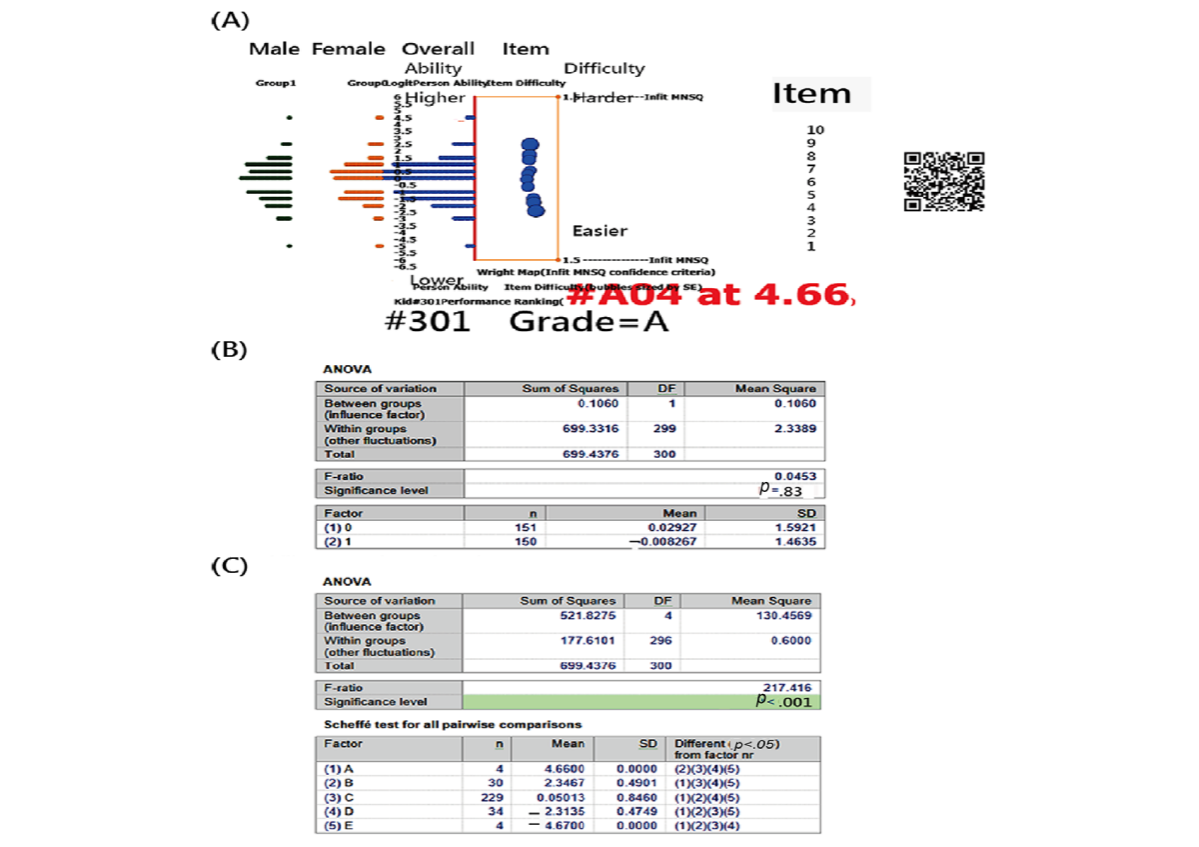
Features of the study sample on Wright map (note: no difference in measures between gender groups was found). (A) Wright map; (B) Ability comparison of gender; (C) Ability comparison of grade.

The Wright in Figure 5 illustrates several findings. First, item difficulties are arranged from harder to easier on the right panel. Second, the middle panel displays person measures distributed from high to low abilities. Third, the left panel shows the display of person measures in groups. Fourth, the bottom panel indicates that there is no significant difference in measures between the 2 groups of males and females (*P*=.85), but a significant difference was found among strata. Finally, based on the grade criteria of person measures (eg, from A to E), GPT301, with measures of 4.66 logits, is classified as grade A, indicating excellent performance in answering 10 items from Taiwan college entrance examinations for the year 2023 (Table 1 and Multimedia Appendix 1) when compared to the normal sample generated by responses under the Rasch model.

**Figure 6:**
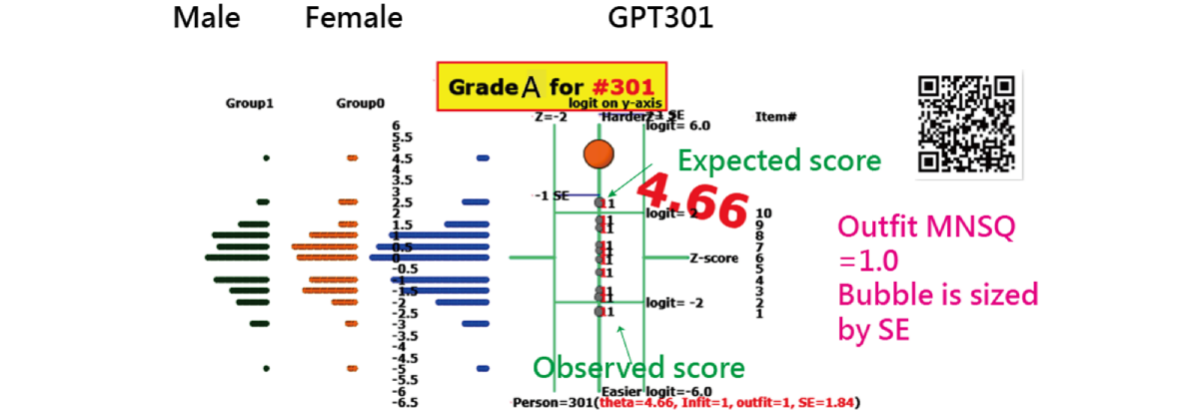
Performance of GPT30 shown on KIDMAP (note: expected scores are vertical with red fonts in the middle and observed scores are vertical with black fonts in the middle). MNSQ: meansquare.

According to Figure 6, the responses of GPT301 are expected within the upper and lower limits (ie, *z* score in item *i* = (observed – expected)/(SD) of item *i*<2.0). The Outfit MNSQs are smaller than 2.0, which indicates that no aberrant responses exist in items [52] (ie, person responses are consistent with Rasch’s expectations). This is because the GPT300 has 100% correct answers to the 10 items from Taiwan college entrance examinations for the year 2023 (Table 1 and Multimedia Appendix 1).

Accordingly, this study confirms the second goal of determining that the ChatGPT’s grade is A when compared to a normal sample.

### Website Dashboards Shown on Google Maps

For readers who wish to manipulate dashboards independently, those QR codes are provided in Figures (or at links [53-57]).

## Discussion

### Principal Findings

The study findings showed that the 10 items displayed progressively more difficulty from easiest to hardest, as indicated by their respective logit scores (–2.43, –1.78, –1.48, –0.64, –0.1, 0.33, 0.59, 1.34, 1.7, and 2.47). Item 5 exhibited DIF between gender groups, with a *P* value of .04. However, item 5 still fits the Rasch model reasonably well, with a *P* value of .61. All items were deemed to fit the Rasch model since their Infit MNSQs were below the threshold of 1.5. There was no significant difference in measures obtained between male and female participants (*P*=.83), but there was a significant difference among ability grades (*P*<.001). Finally, based on its performance, ChatGPT received a grade of A, surpassing grades B to E in other counterparts.

Accordingly, two objectives have been achieved: (1) to demonstrate the use of website Rasch analysis (namely, RaschOnline [44]) and (2) to determine the ChatGPT’s grade compared to a normal sample.

### What This Knowledge Adds to What We Already Knew

ChatGPT has demonstrated accuracy across various data sets such as answering yes-or-no questions from PubMed abstracts, questions on the USMLE, and breast cancer screening and select-all-that-apply prompts [12,58]. While Ha and Yaneva [30] reported low accuracy rates for MCQs, this study found that GPT301 exhibited high accuracy rates for MCQs in 10 items from the 2023 Taiwan college entrance examinations (Table 1 and Multimedia Appendix 1).

According to a study [12], ChatGPT’s performance on the USMLE exceeded a 60% threshold and demonstrated the ability to achieve a passing score equivalent to that of a third-year medical student.

On the other hand, ChatGPT was assessed on all 3 sections of the USMLE: step 1, step 2CK, and step 3 [25,59]. The study findings revealed that ChatGPT achieved or nearly achieved the passing threshold for all 3 examinations without requiring any specialized training or reinforcement.

Past research on medical question answering has predominantly focused on assessing model performance on specific tasks [58]. ChatGPT was rated as a grade of A minor for answering prompts related to Kawasaki disease [3].

A study [60] found that ChatGPT and other assistants hold great potential as useful tools for both patients and health care providers, as they are capable of handling a broad range of assessments from basic fact-based questions to complex clinical queries. Compared to Google’s feature snippet, ChatGPT was able to provide interpretable responses that minimized the risk of causing undue alarm. However, given the nascent stage of this technology, it is crucial for regulators and health care professionals to collaborate in establishing minimum quality standards and educating patients about the limitations of AI assistants [61]. As we consider the transformative impact of these advancements on medical education and research, it is important to recognize the potential benefits and drawbacks of this technology [62].

In terms of accuracy, GPT-4 demonstrated superior performance compared to GPT-3.5, particularly in handling general, clinical, and clinical sentence questions [5]. Moreover, GPT-4 successfully met the passing criteria for the Joint Medical Licensure Examination, affirming its dependability in clinical reasoning and medical knowledge, even in non-English languages [5].

Korn and Kelly [9] have raised concerns about ChatGPT’s reliability and fairness, in line with reports in the popular press regarding misinformation issues. The authors caution that ChatGPT may not always provide accurate information, and there are fears that it could be manipulated to spread false information [10] or produce “deepfakes” [11].

Based on the results of this study, ChatGPT can provide answers to MCQs with an excellent level of accuracy and consistency across the 10 prompts provided. The study suggests that ChatGPT can be a valuable tool for MCQs in English language tests. However, it is essential to exercise caution when using ChatGPT for other forms of English language tests.

Several computer programs, such as WINSTEPS [63], Quest [64], ConQuest [65], RUMM2030 [66], WINMIRA [67], LPCM-Win [68], and R-language Rasch software [69], have been developed to calibrate item and person parameters in Rasch models. However, none of these software packages provide a website Rasch analysis technique that is easily accessible to users and allows for the creation of visual graphs (such as the Wright map, KIDMAP, category probability curves, student outfit plots, and DIF detection), which are commonly used in Rasch analysis.

The website reports generated by RaschOnline provide estimations that are equivalent to those obtained using the Joint Medical Licensure Examination in WINSTEPS [63]. These estimations are like, but more accurate than, those obtained in a previous study [70], which relied on copying and pasting data instead of directly uploading it to the website.

To generate visual graphs, the Rasch model parameters must first be obtained. Then, it is necessary to assess whether the data set meets the requirements for invariant measurement, as depicted in Figures 2, 4, and 5. Additionally, DIF detection is a crucial aspect of Rasch analysis [38,39,71-73], as shown in Figure 3. Providing website access to test results is vital for teachers and students, as demonstrated by the RaschOnline platform [44,50] in this study.

### The Strengths and Features of This Study

In this study, the capacity of ChatGPT was evaluated. The study compared ChatGPT’s responses to 10 items of MCQs using the Wright Map and KIDMAP to compare ChatGPT’s ability with other simulated participants in Rasch analysis.

The study found that ChatGPT has the potential to improve the English learning process, and it demonstrated the feasibility of using ChatGPT for other types of participants (eg, patients) with symptoms commonly encountered in clinical settings.

According to this study, (1) ChatGPT has an excellent level of ability to answer MCQs in English examinations, and (2) the effectiveness of ChatGPT is determined by a grade A with 4.66 logits. We suggest that the methods and visualizations used in this study can be replicated in future research using RaschOnline [34].

The distinct features of this study include the following: (1) the data were analyzed using RaschOnline [44], a tool based on Rasch RSM. This enabled the use of visualizations, such as Wright Map with groups, DIF using forest plots, and KIDMAP, to display item features and person responses. These visualizations had not been previously demonstrated in the literature and can be accessed on RaschOnline for more information and demonstrations; (2) using objective measurement through Rasch analysis to analyze responses, ChatGPT has demonstrated a high level of proficiency in answering MCQs; (3) the efficacy of ChatGPT has been established; however, future evaluations of ChatGPT’s performance on open-ended questions must be conducted with caution due to potential bias in judges’ leniency and severity.

### Limitations and Directions for Future Studies

This study has certain limitations that may motivate further research. The first concern is that the data were generated using Rasch simulation responses, as shown in Multimedia Appendix 2, based on the Rasch model [21]. The real and simulated responses to the 10 items were compared.

Second, RashOnline [34] has clearly been shown to be applicable in use [25] rather than traditional professional statistical software (eg, WINSTEPS [63], Quest [64], ConQuest [65], RUMM2030 [66], WINMIRA [67], LPCM-Win [68], and R-language Rasch software [69]), and further research should be conducted to determine whether the visualizations generated using Google Maps in RaschOnline are more straightforward and easier to use for general researchers.

Third, on the basis of the study sample size (n=300 in this study), it is not possible to draw reliable and valid conclusions. For a reliable and accurate assessment, there is a need for a larger sample size in future research.

Fourth, in this study, only 10 items were used. A test or assessment that contains more items will be more reliable. To assess ChatGPT’s ability, more items will be needed in the future.

Fifth, in the case of an OE assessment [3,14], ChatGPT’s ability is dependent upon the judge’s leniency and severity. The results of the OE assessment reveal that the 2 judges have distinctly different attitudes toward the responses provided by the ChatGPT. The conditions of leniency and severity in the assessment of ChatGPT should be stricter in the future.

Finally, although AI technologies, such as ChatGPT, have demonstrated their potential in assisting medical decision-making in certain domains [3,12,14,58], such as identifying particular ailments or interpreting medical images, they are not sufficiently advanced to replace physicians in intricate diagnoses or treatment planning. Nevertheless, as technology advances, it is conceivable that AI may play a more prominent role in health care decision-making in the future.

### Conclusions

This paper evaluates the effectiveness of ChatGPT in answering MCQs using Rasch analysis. The study used RaschOnline to assess ChatGPT’s capabilities and compared its performance to a normal sample.

The findings of this study reveal that ChatGPT’s ability to answer MCQs is graded as A, indicating excellent performance. The study showcases the use of website Rasch analysis and highlights ChatGPT’s remarkable proficiency in addressing English test MCQs for the year 2023 on Taiwan college entrance examinations.

While AI technologies have displayed promising potential in assisting medical decision-making, they are not yet advanced enough to replace medical doctors in complex diagnoses or treatment planning. However, with the continuous evolution of technology, AI has the potential to play an increasingly significant role in health care decision-making in the future.

## Appendix

Multimedia Appendix 1 Ten items from Taiwan college entrance examinations for the year 2023.

Multimedia Appendix 2 Data used in this study.

Multimedia Appendix 3 How to conduct this study.

## Abbreviations

AI: artificial intelligence
DID: distribution of item difficulty
DIF: differential item functioning
ICC: item characteristic curve
MCQ: multiple-choice question
MNSQ: meansquare
OE: open-ended
RSM: rating scale model
USMLE: United States Medical Licensing Examination

## Data Availability

All data generated or analyzed during this study are included in this published article and its Multimedia Appendices.
