# Supplementary material for: Assessing ChatGPT’s Capability for Multiple Choice Questions Using RaschOnline: Observational Study
Source: JMIR Form Res. 2024 Aug 8;8:e46800. doi: 10.2196/46800 (PMC11346125; doi:10.2196/46800)

財團法人大學入學考試中心基金會

112學年度學科能力測驗試題

英文考科

請於考試開始鈴響起，在答題卷簽名欄位以正楷簽全名

—作答注意事項—

考試時間：100分鐘

作答方式：

- 選擇題用 2B 鉛筆在「答題卷」上作答；更正時以橡皮擦擦拭，切勿使用修正帶（液）。
- 除題目另有規定外，非選擇題用筆尖較粗之黑色墨水的筆在「答題卷」上作答；更正時，可以使用修正帶（液）。
- 考生須依上述規定劃記或作答，若未依規定而導致答案難以辨識或評閱時，恐將影響成績。
- 答題卷每人一張，不得要求增補。

選擇題計分方式：

- 單選題：每題有 n 個選項，其中只有一個是正確或最適當的選項。各題答對者，得該題的分數；答錯、未作答或劃記多於一個選項者，該題以零分計算。
- 多選題：每題有 n 個選項，其中至少有一個是正確的選項。各題之選項獨立判定，所有選項均答對者，得該題全部的分數；答錯 k 個選項者，得該題 $\frac{n-2k}{n}$ 的分數；但得分低於零分或所有選項均未作答者，該題以零分計算。

第壹部分、選擇題（占62分）

一、詞彙題（占10分）

說明：第1題至第10題為單選題，每題1分。

1. The bus driver often complains about chewing gum found under passenger seats because it is _____ and very hard to remove.
(A) sticky　(B) greasy　(C) clumsy　(D) mighty

2. Jesse is a talented model. He can easily adopt an elegant ______ for a camera shoot.
(A) clap　(B) toss　(C) pose　(D) snap

3. In order to draw her family tree, Mary tried to trace her ______ back to their arrival in North America.
(A) siblings　(B) commuters　(C) ancestors　(D) instructors

4. Upon the super typhoon warning, Nancy rushed to the supermarket—only to find the shelves almost ______ and the stock nearly gone.
(A) blank　(B) bare　(C) hollow　(D) queer

5. Even though Jack said “Sorry!” to me in person, I did not feel any ______ in his apology.
(A) liability　(B) generosity　(C) integrity　(D) sincerity

6. My grandfather has astonishing powers of ______. He can still vividly describe his first day at school as a child.
(A) resolve　(B) fraction　(C) privilege　(D) recall

7. Recent research has found lots of evidence to ______ the drug company’s claims about its “miracle” tablets for curing cancer.
(A) provoke　(B) counter　(C) expose　(D) convert

8. Corrupt officials and misguided policies have ______ the country’s economy and burdened its people with enormous foreign debts.
(A) crippled　(B) accelerated　(C) rendered　(D) ventured

9. As a record number of fans showed up for the baseball final, the highways around the stadium were ______ with traffic all day.
(A) choked　(B) disturbed　(C) enclosed　(D) injected

10. Studies show that the ______ unbiased media are in fact often deeply influenced by political ideology.
(A) undoubtedly　(B) roughly　(C) understandably　(D) supposedly

二、綜合測驗（占10分）

說明：第11題至第20題為單選題，每題1分。

第 11 至 15 題為題組

In certain forests, when you look up you will see a network of cracks formed by gaps between the outermost edges of tree branches. It looks like a precisely engineered jigsaw puzzle, each branch growing just perfectly so it almost, __11__, touches the neighboring tree. This beautiful phenomenon is called crown shyness.

Scientists have been discussing crown shyness since the 1920s, proposing __12__ explanations for the phenomenon. Some researchers point out that as trees often grow close together, treetops can easily collide and break when swayed by the wind. In order to protect their branches from breakage, trees maintain "shyness gaps"—spaces large enough to prevent them from touching their neighbors.

Other scientists suggest that plants, like animals, __13__ resources—nutrients, water, space, and light. In forested areas with thick tree crowns, there is intense struggle for these resources. Gaps in the treetops resulting from crown shyness may allow trees to increase their __14__ light and enhance photosynthesis. Additionally, by having branches that do not touch those of their neighbors, trees may be able to limit the spread of leaf-eating insects, and potentially also the transmission of diseases from tree to tree. __15__ decades of investigation, there is no consensus on exactly what causes the beautiful and mysterious phenomenon of crown shyness.

11.	(A) in no time	(B) by all means	(C) but not quite	(D) and pretty much
12.	(A) universal	(B) productive	(C) conventional	(D) multiple
13.	(A) get over	(B) compete for	(C) give way to	(D) make up for
14.	(A) reliance on	(B) exposure to	(C) sensitivity to	(D) reflection on
15.	(A) For	(B) Besides	(C) Despite	(D) Concerning

第 16 至 20 題為題組

Gravity has been at the top of the science agenda since the start of Mars missions. In the earlier days of space travel, scientists tried to overcome the force of gravity so that a rocket could shoot __16__ Earth's pull in order to land humans on the moon. Today, they are more interested in how reduced gravity affects the astronauts' __17__ condition.

Our bodies have evolved to exist within Earth's gravity (1 g), not in the weightlessness of space (0 g) or the microgravity of Mars (0.3 g). When on Earth, we have more fluids in our lower body because they are pulled down by Earth's gravity. However, with the absence of gravity in the outer space, our body fluids __18__, shifting toward the upper body and the head. As a result, the astronauts have swollen, puffy faces, very much resembling that of the round-headed Charlie Brown in the famous comic strip. This "Charlie Brown effect" will be more __19__ when the astronauts go on their Mars missions, which will take about three years to complete, much longer than missions to the moon. Moreover, the effect is often __20__ space motion sickness, headaches, and nausea. Such a syndrome is considered the top health risk for the astronauts, and scientists are still trying to figure out how it may be prevented.

16.	(A) back to	(B) free of	(C) long before	(D) straight on
17.	(A) physical	(B) perceptual	(C) mental	(D) external
18.	(A) redistribute	(B) redistributed	(C) redistributing	(D) being redistributed
19.	(A) contagious	(B) unusual	(C) severe	(D) aggressive
20.	(A) varied with	(B) brought about	(C) transferred from	(D) accompanied by

三、文意選填（占10分）

說明：第21題至第30題為單選題，每題1分。

第 21 至 30 題為題組

Water makes up more than half of our body weight. To sustain this amount of fluid in our bodies, plain water is considered our best choice, for it contains no sugar and no calories. Yet, is water always the healthiest drink we can __21__? Well, it depends on who and where we are, and what we're doing.

Obviously, a physically __22__ person with an outdoor job under the sun will need to drink more fluid than a desk-bound person who lives and works in an air-conditioned place. But there's more to it than that. When a person sweats, he loses water and salt, so he needs to replace both. Replacing lost fluid with just plain water means the body has too much water and not enough salt. To __23__ things out, the body will get rid of water by producing urine. For this reason, milk can actually be more __24__ than drinking water. Milk naturally contains salt and lactose, a sugar which the human body needs in small amounts to help stimulate water __25__. Coconut water, which contains salt and carbohydrates, is also more functional than water at restoring and maintaining a normal fluid __26__ after exercise.

For the average person, however, water remains a very good __27__ for keeping hydrated—if you know how to drink it. The secret is: Never wait until the body is telling you you're thirsty, since there must already have been significant changes in your body for it to eventually __28__ your consciousness. At that point, it might be well past the best moment to take in fluid. Also, drinking a lot of liquid in one go can cause more water to __29__ the body quickly and come out as urine. To __30__ this, you need to drink water throughout the day to maintain your hydration levels.

(A) absorption	(B) active	(C) alert	(D) combat	(E) option
(F) effective	(G) even	(H) status	(I) pass through	(J) reach for

四、篇章結構（占8分）

說明：第31題至第34題為單選題，每題2分。

第 31 至 34 題為題組

Have you ever thought of “coloring” the names of the days of the week? When you listen to someone speaking, do you see a rainbow of colors? Or perhaps Mozart’s music tastes like an apple pie to you? If so, it is very likely that you have synesthesia.

Synesthesia is a condition in which people’s senses intermix. In some cases, people with synesthesia may experience colors when they hear, read, or even think of letters and numbers. In others, words can trigger a real sensation of taste on their tongue.

__31__ In the early 1990s, however, scientists noticed that synesthetic colors do not change over time. When asked what color is evoked by a letter or number, synesthetic people would persistently give the same answer even if tested months or years apart. __32__ The most compelling support, however, comes from brain scans, which show that color processing areas in the brain light up when these people listen to certain words.

Is synesthesia genetically inherited or acquired after birth? Scientists agree that synesthesia has a genetic basis, because it frequently runs in families. But an actual synesthesia gene (or genes) has not been identified yet. __33__ For example, the flavors people with taste-word synesthesia experience are usually childhood flavors, such as chocolate or strawberries. Also, people with color-music synesthesia more often than not have had early musical training.

Once thought to be extremely rare, synesthesia is now found to affect about one to four percent of the population. __34__ As is often observed, most of us tend to associate lower notes with darker colors and higher notes with brighter colors. Researchers further point out that in most people synesthesia is active only during the first months of their infancy, while this ability remains forever in certain individuals.

(A) This consistency serves as a proof that synesthesia is real.

(B) Meanwhile, environmental influences seem to shape a person’s synesthesia.

(C) People with synesthesia used to be accused of making their experiences up.

(D) Some studies even show that people may all be synesthetic to some degree.

五、閱讀測驗（占 24分）

說明：第35題至第46題為單選題，每題2分。

第 35 至 38 題為題組

When did people first experience the joy of the hula hoop? Although the term did not emerge until the 18th century, toy hoops twirled around the waist, limbs, or neck can be traced back to ancient times. As early as 1000 BC, Egyptian children played with hoop toys of dried grapevine. They threw, jumped, and slung them around their bodies as we do today. They also struck them with sticks to roll them down the road. Hoop rolling was also popular in ancient Greece. Their hoops, often made of metal, were not merely toys for Greek children but served as exercise devices as well.

In the 14th century, hoops were popular as a form of recreation in Great Britain. The craze for hoops even resulted in dislocated backs and heart attacks, according to medical records. The term "hula," however, did not enter the English language until the 1700s, when British sailors first witnessed hula dancing in the Hawaiian Islands. Though no hoops were used, the movements of the ritual dances looked very similar to those in hooping. Thus "hula" and "hoop" came together, resulting in the term "hula hooping."

Hoops spun their way through the cultures of pre-colonial America as well. Often considered as representing the circle of life, hoops featured prominently in the ritual dances of Native Americans. Dancers used small reed hoops as symbolic representations of animals such as eagles or snakes. With very rapid movements, they used the hoops to construct the symbolic forms around their bodies.

The hula hoop gained international popularity in the late 1950s, when a plastic version was successfully marketed by California's Wham-O toy company. Twenty-five million plastic hoops were sold in less than four months. The hula hoop "fad" is still going strong today.

35. What question does the passage answer?
    (A) How was the word "hula-hooping" derived?
    (B) Why did Wham-O start making hula hoops?
    (C) Where did Hawaiian hula dancing come from?
    (D) What was the favorite toy of ancient Egyptian kids?

36. Which of the following statements is true about use of the hoop in history?
    (A) The British used it for medical purposes.
    (B) Native Americans used it to train animals.
    (C) Ancient Greeks used it as a tool for workout.
    (D) Hawaiian dancers used it to represent the circle of life.

37. Which of the following is **NOT** mentioned as a way of enjoying hula hoop fun?
    (A) Striking. (B) Twirling. (C) Spinning. (D) Kicking.

38. According to the passage, what materials have been used for making hoops?
    (A) reed, grapevine, bamboo, plastic
    (B) reed, grapevine, plastic, metal
    (C) reed, bamboo, plastic, animal skin
    (D) grapevine, plastic, metal, animal skin

第 39 至 42 題為題組

When you enjoy your morning cup of tea, you are probably not aware that those tea leaves can mean injury, or even death, for Asian elephants roaming Indian tea gardens.

In the Indian state of Assam, growing numbers of tea farms are destroying the Asian elephant's habitats and endangering their population. Much of the forest land where tea is grown in Assam is flat and thus farmers must dig drainage trenches to prevent water from accumulating and hurting the shrubs. The trenches, however, can be death traps for the elephants. Since the elephants need to use tea plantations as landmarks when navigating forests, they almost inevitably have to move through the farms. Moreover, because there are fewer humans around, pregnant females often use tea-growing areas as safe shelters to give birth. But baby elephants, not used to negotiating rough ground, may easily fall into the trenches and get hurt; and once injured, they might not be able to climb out. When mothers try to dig their babies out, both may be trapped and smothered by thick mud. Furthermore, elephants are known to resist leaving their sick or dying behind, and a herd may linger at a trench with a trapped baby for hours, reluctant to move on until all hope is lost.

Is it possible for elephants to coexist with the prosperous tea business? Elephant Friendly Tea is an organization that takes the initiative to make **it** possible. The organization encourages consumers to choose brands that take precautions to protect elephants and has set up a certification program to reward tea growers who are doing it right. Until now, only smaller tea brands have been certified, but awareness is growing. The organization believes that people may be motivated to buy elephant-friendly brands when they know more about the risk tea can pose to these endangered animals.

39. Why do farmers in Assam dig trenches in tea gardens?
    (A) To protect tea trees. (B) To trap elephants.
    (C) To expand tea farms. (D) To mark boundaries of tea gardens.

40. Why are baby elephants easily injured in the Assam tea gardens according to the passage?
    (A) They cannot find a safe shelter when climbing out of the trenches.
    (B) They cannot locate the landmarks while trying to navigate forests.
    (C) They are trapped by the sharp branches of the tea trees.
    (D) They have difficulties moving around the uneven fields.

41. Which of the following statements about elephants and the tea gardens is true according to the passage?
    (A) The elephants use the trenches to roam around the tea gardens.
    (B) The fast growth of the tea gardens destroys the elephants' food source.
    (C) Elephants are unwilling to leave their injured members behind in the tea gardens.
    (D) Pregnant elephants avoid delivering babies in the tea gardens for fear of being disturbed.

42. What does "**it**" in the last paragraph refer to?
    (A) To certify elephant-friendly trenches and organizations.
    (B) To reward tea growers for protecting the environment.
    (C) To encourage consumers to choose high-quality brands.
    (D) To create a win-win situation for elephants and tea farms.

第 43 至 46 題為題組

Situated off the coast of Tanzania and washed by the warm, clean waters of the Indian Ocean, Zanzibar is a tropical archipelago comprised of several scattered islands. This popular beach destination is now famous for its white sand beaches, slender palms, and turquoise seas. But few people know that in the past, control of Zanzibar meant access to unimaginable wealth.

From ancient times, Zanzibar has been a trading hotspot, thanks to its location on the trade route between Arabia and Africa. Traders from Asia had already visited the islands 900 years before the arrival of its first permanent settlers from the African mainland (around 1000 AD). In the 8th century, Persian merchants built settlements here, which grew over the next four centuries into their trading posts. Between the 12th and 15th centuries, trade increased between Arabia, Persia, and Zanzibar, bringing the archipelago both wealth and power.

During the Age of Exploration, commerce in Zanzibar quickly boomed, largely due to the rise of the spice trade. At the close of the 15th century, Europeans' craze for spices gave rise to the Spice Route, a network of sea lanes joining Europe with the Far East, where most spices came from. In 1498, Portuguese explorer Vasco da Gama made the first sea voyage to India, via the southernmost tip of Africa. In 1499, he arrived at Zanzibar, an archipelago sitting at the crossroads of the Spice Route. The islands soon attracted traders from different lands. Hundreds of ships sailing the Spice Route docked here, bringing spices and goods for transaction, and Zanzibar became one of the biggest trading centers in the world.

Since the 16th century, Zanzibar has come under the rule of the Portuguese, the Arabians, and then the British, each leaving a mark on the place. The paths of various religions also crossed here: Muslims have lived peacefully with Christians and Buddhists on the islands for centuries. The unique cultural intersections, scented with the aroma of cloves, vanilla, and cinnamon floating in the air, give these jewels on the Indian Ocean an amazing charm that goes far beyond tropical beach fun.

43. Which of the following is true about the earliest traders in Zanzibar?
(A) The earliest traders arrived around 900 AD.
(B) Most of the earliest merchants came from Africa.
(C) Asian merchants arrived centuries before the African settlers.
(D) Traders from Persia settled down permanently around 1000 AD.

44. According to the passage, where is Zanzibar most likely located on the following map?

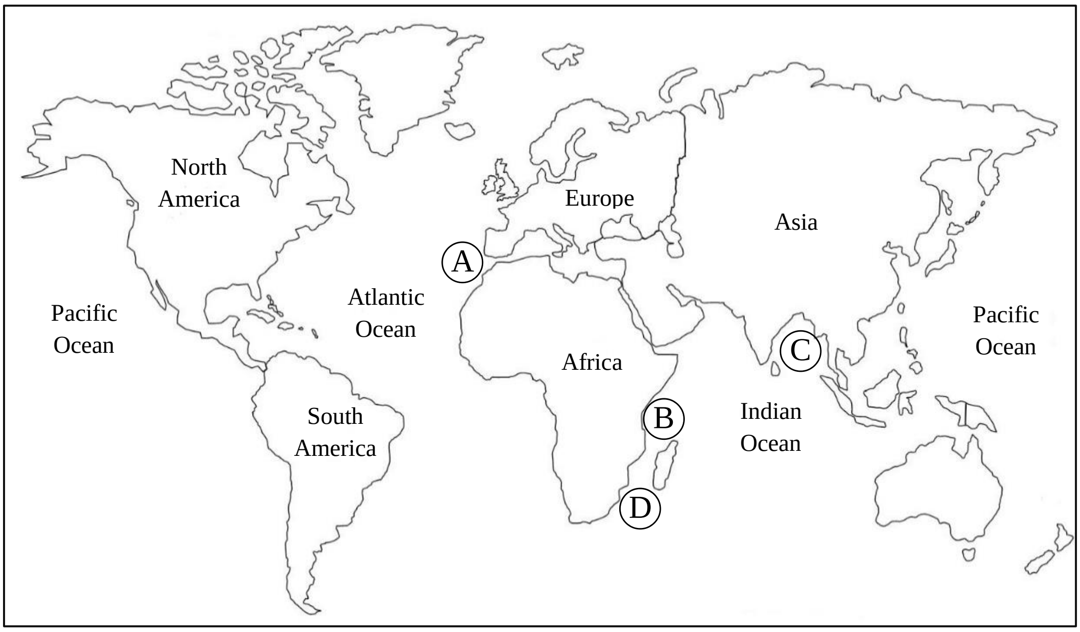

(A) A (B) B (C) C (D) D

45. Which of the following can be inferred from the passage about Zanzibar?
(A) For centuries, Zanzibar has been a heaven for beach lovers.
(B) Cloves, vanilla, and cinnamon are common spices in Zanzibar.
(C) Besides spices, Zanzibar is well known for a great variety of jewelry.
(D) Vasco da Gama was Zanzibar’s first foreign ruler during the Age of Exploration.

46. Which set of words is used in the passage to refer to Zanzibar?
(A) islands, settlements, posts, crossroads
(B) islands, posts, jewels, destination
(C) archipelago, islands, jewels, destination
(D) archipelago, settlements, paths, islands

第貳部分、混合題（占10分）

說明：本部分共有1題組，每一子題配分標於題末。限在答題卷標示題號的作答區內作答，並以規定用筆作答。

Even if you’re not a vegetarian, there’s a good chance you’ve heard of plant-based meat. These meat substitutes are often advertised as beneficial for the environment. **But do the customers really like them?** Read the following chatroom discussions about GreenBurger, a product using alternative-meat patties.

(A) Olivia

That’s a firm NO! I tried one when I was a prep cook for Next Level Burgers…tasted awful, just like mashed peas gone to rot.

(B) Smith

I haven’t tried them. I just don’t see the point. They are heavily processed with more calories and sodium than meat and are more expensive too! Unless you are a strict vegetarian and eschew meat for ethical reasons, meat is surely a better deal.

(C) Mika

Not really. It tastes too much like actual meat. If I wanted to eat a dead cow, I would go for real burgers. Being vegan means I try to stick to plant food. But this is just not something that will ever interest me.

(D) Thomas

GreenBurger just tastes “beany” to me, so I think that whatever form it comes in, it works better in recipes that would usually contain beans, such as in a rice bowl (as ground meat substitute). That being said, I’m definitely a carnivore and prefer meat, but I don’t see anything wrong with choosing one of the fakey-fakey meaty things once in a while—just for a change.

(E) Rico

I finally tried one last week. It didn’t taste that bad, but I was surprised to find it had 40 more calories than the meat version! Isn’t it supposed to be a healthy choice?

(F) Linda

I liked it fine. I did NOT like the price, though. Hopefully that will come down. I’m trying to eat less beef.

(G) Ablo

Yes! My whole family does. We’ve been mostly vegan 90% of the time for the last 6 years and we cannot thank GreenBurger enough! Especially when a burger craving hits…a GreenBurger does the trick. I’ve tried all kinds of vegan “burgers,” and GreenBurgers are closer to what I’m looking for. In fact, I can have them every day—vegan or not!

(H) Alexander

Well, they taste good if you add plenty of spices and sauces. Good for you? No, not at all. They’re highly processed and contain no vegetables. They also contain a very large amount of processed coconut oil, which is even worse than lard. It’s a killer for sure if eaten regularly. So why risk your life?

47-48 請從文章中找出最適當的單詞（word）填入下列句子空格中，並視語法需要做適當的字形變化。**每格限填一個單詞（word）**。（填充，4分）

On the whole, the chatroom discussions about GreenBurger mainly focus on the issues of ___47___, price, and ___48___ concerns.

49. From (A) to (H) in the above chatroom discussions, which ones show that GreenBurger can be a choice for the chatroom participants themselves **only under certain circumstances**? （多選題，4分）

50. Which **phrase** in the chatroom discussions means "serves the purpose" or "works well"?（簡答，2分）

____

背 面 尚 有 試 題

第參部分、非選擇題（占 28分）

說明：本部分共有二大題，請依各題指示作答，答案必須寫在「答題卷」標示題號之作答區內，作答時不必抄題。

一、中譯英（占 8分）

說明：依題號將以下中文句子譯成正確、通順、達意的英文。每題4分，共8分。

1. 歷史一再證明，戰爭會造成極為可怕的災難。
2. 避免衝突、確保世界和平應該是所有人類追求的目標。

二、英文作文（占 20分）

說明：依提示寫一篇英文作文，文長至少120個單詞（words）。

提示：隨著社群媒體的普及，表情符號（emoji）的使用也極為普遍。請參考下列表情符號，寫一篇英文作文，文分兩段。第一段說明人們何以喜歡使用表情符號，並從下列的表情符號中舉一至二例，說明表情符號在溝通上有何功能。第二段則以個人或親友的經驗為例，討論表情符號在訊息表達或解讀上可能造成的誤會或困擾，並提出可以化解的方法。

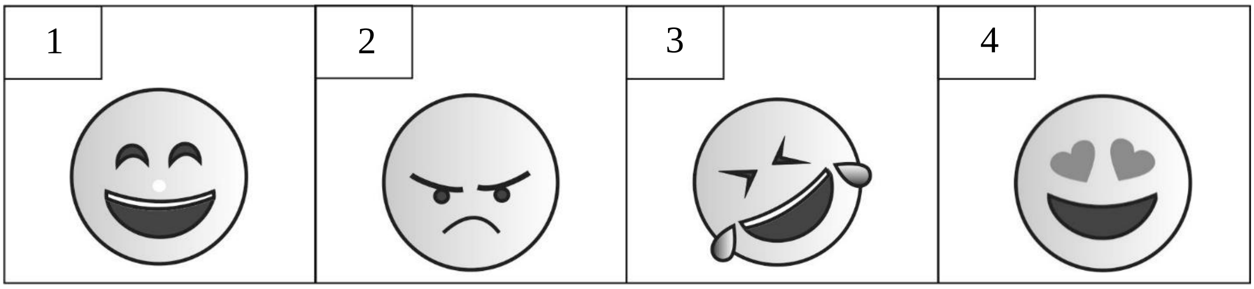

Supplement: Multimedia Appendix 1 [file formative_v8i1e46800_app1.pdf]
