# Supplementary material for: Assessing ChatGPT’s Capability for Multiple Choice Questions Using RaschOnline: Observational Study
Source: JMIR Form Res. 2024 Aug 8;8:e46800. doi: 10.2196/46800 (PMC11346125; doi:10.2196/46800)
Supplement: Multimedia Appendix 3 [file formative_v8i1e46800_app3.pdf]

How to conduct this study

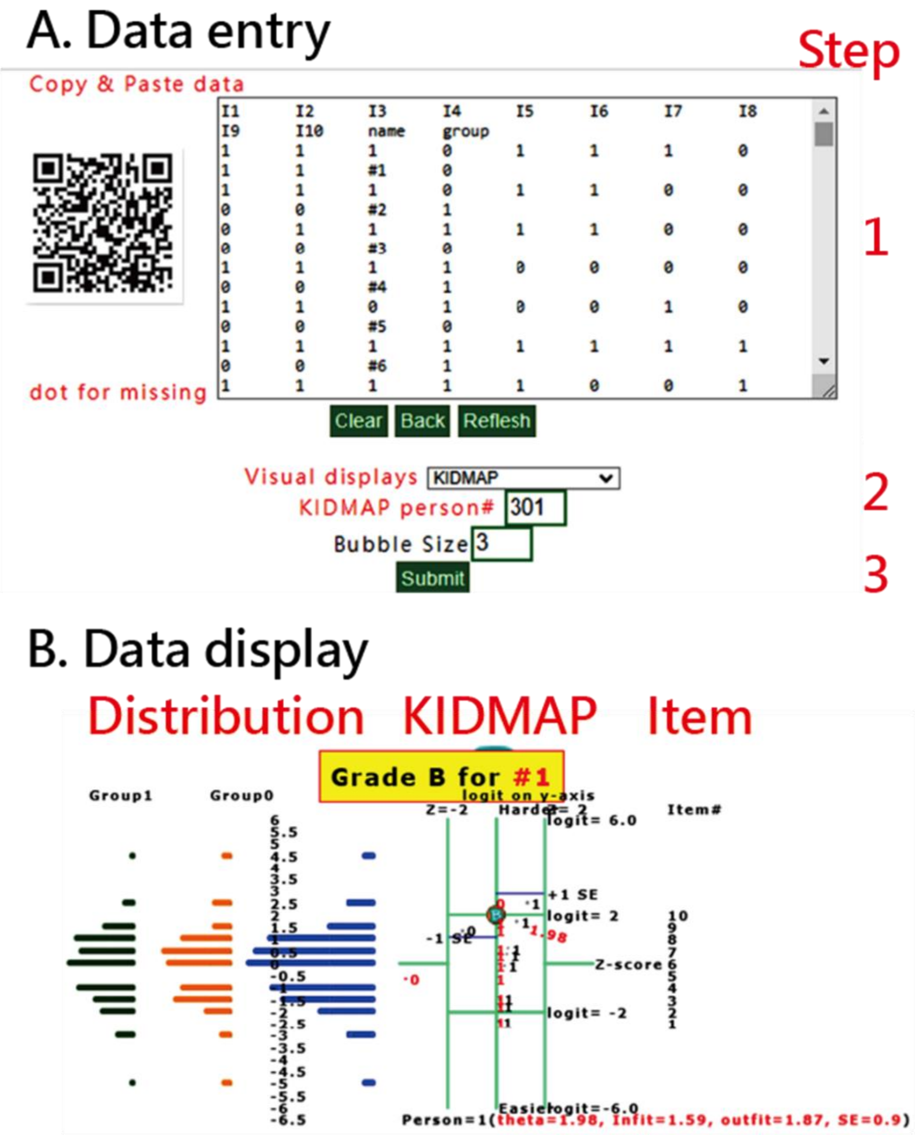

Figure 1 How to execute RaschOnline with the example of KIDMAP (note. 1. Data are copied and pasted to the box frame; 2. Visual presentation is selected; 3. submission icon is clicked to generate results)

https://www.raschonline.com/raschonline/raschrsm.asp?covid=04

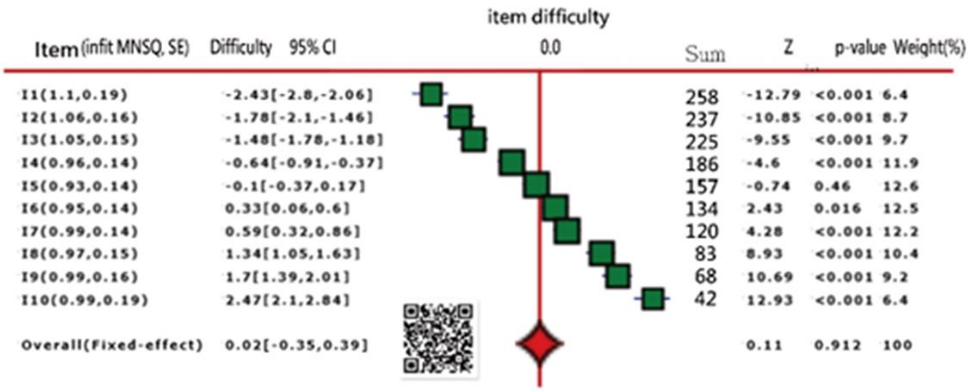

Figure 2 Distribution of item difficulties used in this study

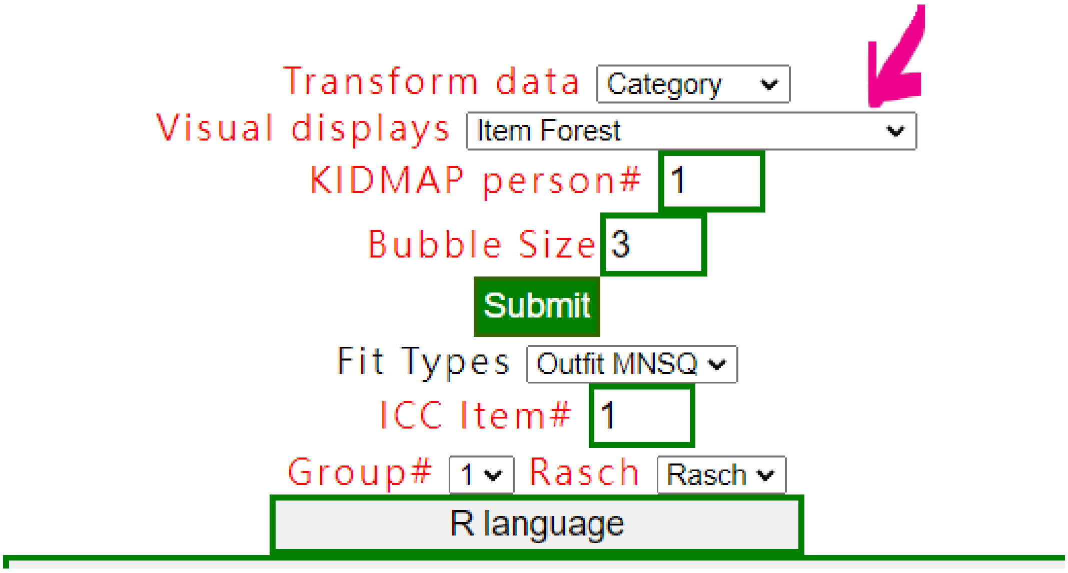

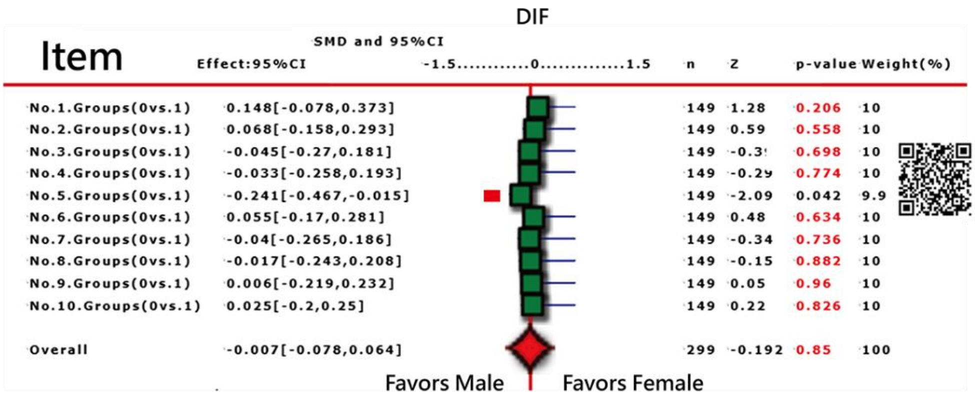

Figure 3 DIF analysis of the ten items in this study (note. item 5 exhibits a small DIF effect with p=0.042<0.05)

https://www.raschonline.com/raschonline/raschrsm.asp?covid=07

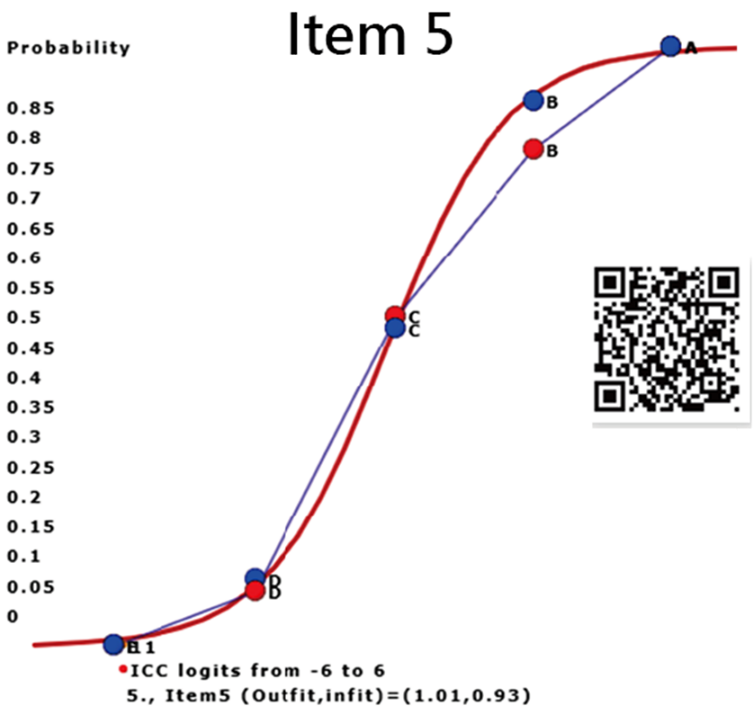

Strata_raw score item=5

Strat	Sum	n	Mean	Expected	Variance
A_1(>3.5)	4	4	1	4	0
B_2(>1.0)	25	30	0.83	27.39	2.35
C_3(>-1.5)	125	229	0.55	121.99	48.72
D_4(>-4.0)	3	34	0.09	3.61	3.18
E_5(<=-4.0)	0	4	0	0	0
ChSQ=	2.74df=		4	prob.=	0.61

]

Figure 4 ICC of item 5 fits to the Rasch model (p=0.61)

https://www.raschonline.com/raschonline/raschrsm.asp?covid=05

A. Wright Map

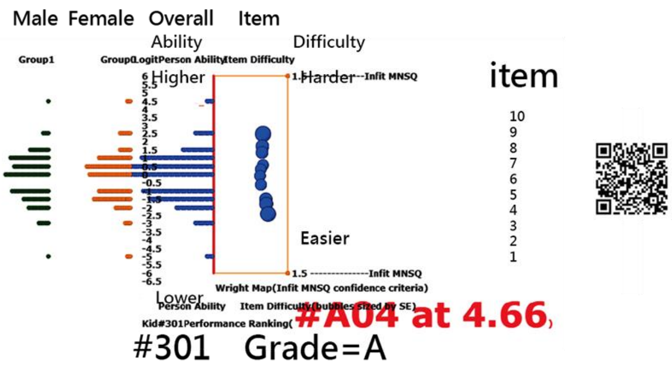

B. Ability comparison of Gender

ANOVA

Source of variation	Sum of Squares	DF	Mean Square
Between groups (influence factor)	0.1060	1	0.1060
Within groups (other fluctuations)	699.3316	299	2.3389
Total	699.4376	300	

F-ratio	0.0453
Significance level	P = 0.832

Factor	n	Mean	SD
(1) 0	151	0.02927	1.5921
(2) 1	150	-0.008267	1.4635

C. Ability comparison of Grade

ANOVA

Source of variation	Sum of Squares	DF	Mean Square
Between groups (influence factor)	521.8275	4	130.4569
Within groups (other fluctuations)	177.6101	296	0.6000
Total	699.4376	300	

F-ratio	217.416
Significance level	P < 0.001

Scheffé test for all pairwise comparisons

Factor	n	Mean	SD	Different (P<0.05) from factor nr
(1) A	4	4.6600	0.0000	(2)(3)(4)(5)
(2) B	30	2.3467	0.4901	(1)(3)(4)(5)
(3) C	229	0.05013	0.8460	(1)(2)(4)(5)
(4) D	34	-2.3135	0.4749	(1)(2)(3)(5)
(5) E	4	-4.6700	0.0000	(1)(2)(3)(4)

Figure 5 Features of the study sample on Wright map (note. no difference in measures between gender groups was found)

https://www.raschonline.com/raschonline/raschrsm.asp?covid=13

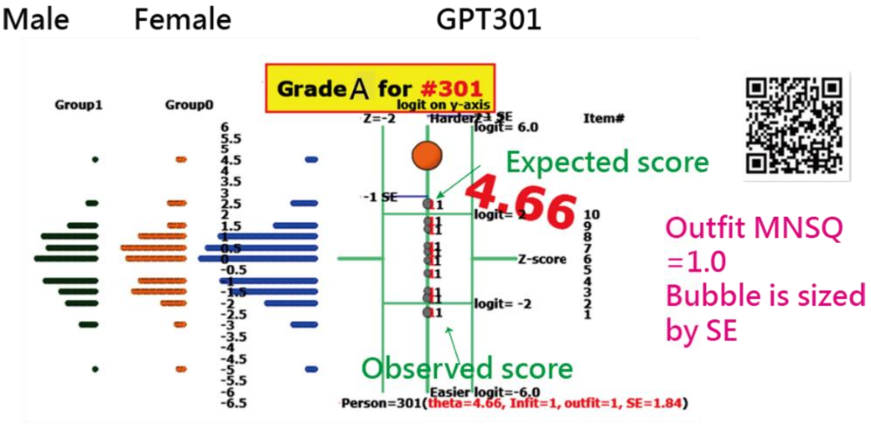

Figure 6 Performance of GPT30 shown on KIDMAP (note. expected scores are vertically with red fonts in the middle and observed scores are vertically with black fonts in the middle)

https://www.raschonline.com/raschonline/raschrsm.asp?covid=04
